# Feasibility, acceptability and potential effects of internet-based cognitive behavioural therapy for prolonged grief

**DOI:** 10.1016/j.invent.2026.100963

**Published:** 2026-06-17

**Authors:** F. Berglund, R. Eklund, P.A. Boelen, E. Hven, L. Ciardella, Y. Rugfelt, V. Kaldo, M. Bragesjö, J. Sveen

**Affiliations:** a Department of Women's and Children's Health, Uppsala University, Uppsala University Hospital, 751 85, Uppsala, Sweden; b Department of Clinical Psychology, Faculty of Social and Behavioural Sciences, Utrecht University, P.O. Box 80140, Utrecht, 3508, the Netherlands; c ARQ National Psychotrauma Centre, Nienoord 5, Diemen, 1112, the Netherlands; d Foundation ARQ Centrum’45, Nienoord 5, Diemen, 1112, the Netherlands; e Primary Care and Health, Region Uppsala, 751 85, Uppsala, Sweden; f Centre for Psychiatry Research, Department of Clinical Neuroscience, Karolinska Institutet, 113 64, Stockholm, Sweden; g Stockholm Health Care Services, Region Stockholm, 104 31, Stockholm, Sweden; h Department of Psychology, Faculty of Health and Life Sciences, Linnaeus University, 351 95, Växjö, Sweden

**Keywords:** Prolonged grief, Online treatment, Bereavement, Cognitive behavioural therapy

## Abstract

The death of a loved one is associated with an increased risk of developing severe mental health complications such as prolonged grief disorder (PGD), post-traumatic stress disorder (PTSD) and depression. Research supports the efficacy of cognitive-behavioural therapies (CBT), delivered online or face-to-face, in treating post-loss mental health problems. However, evidence-based treatments for bereaved adults remain scarce within the Swedish healthcare system.

The main aim of this study was to evaluate the feasibility and acceptability of grief-focused internet-delivered CBT (ICBT) for PGD in Swedish bereaved adults, using an uncontrolled multiple-methods design. A secondary aim was to assess the potential effect on symptoms of PGD, PTSD and depression. Fourteen participants who met criteria for probable PGD were enrolled in the study and received access to the online treatment for eight weeks. Ten participants completed post-treatment interviews and self-report questionnaires about their experiences of the treatment. Self-reported symptom-levels of PGD, PTSD and depression were assessed at baseline, mid-, post-treatment and at four-month follow-up.

Overall, the grief-focused ICBT was perceived as helpful and emotionally engaging. Most participants reported satisfaction with the intervention. Reductions were observed in symptoms of PGD, PTSD and depression, although these findings should be interpreted as preliminary due to the small sample size and limited statistical power. Overall, the results suggest that grief-focused ICBT is a feasible and acceptable treatment for bereaved adults. A fully powered randomised controlled trial is warranted to establish the efficacy of the treatment.

## Highlights

• ICBT for prolonged grief is a feasible and acceptable treatment for bereaved adults.
• Participants were interviewed about their experiences at post-treatment.
• Participants reported high satisfaction rates.
• Preliminary reductions were found in prolonged grief, PTSD and depressive symptoms.

## 1 Introduction

The death of a loved one is one of the most common potentially traumatic events occurring in people's lifetime (Benjet et al., 2016). Common reactions following the death of a family member or a friend include separation distress, disbelief, anxiety, sadness and depressive symptoms (Shear, 2015). For most bereaved, grief reactions remain low or gradually decrease over time (Nielsen et al., 2019). However, for an important subset, intense grief reactions prevail and cause clinically significant suffering. Among individuals bereaved by natural causes (such as old age or illness) (Lundorff et al., 2017), up to 10% develop clinically relevant prolonged grief reactions. This proportion increases to as much as 50% among those bereaved by unexpected and unnatural causes, such as suicide, homicide, and accidents (Djelantik et al., 2020). Moreover, the comorbidity between prolonged grief reactions and other mental health complications is high, including post-traumatic stress disorder (PTSD) and depression (Komischke-Konnerup et al., 2021).

Prolonged grief disorder (PGD) has recently been recognised as a formal classification in both the ICD-11 (World Health Organization [WHO], 2019) and DSM-5-TR (American Psychiatric Association [APA], 2022). This condition is characterised by pervasive and debilitating grief reactions after the death of a family member or a close friend, that interfere with daily functioning. National prevalence data of PGD in Sweden are not available, however, in general populations the point prevalence is estimated at 1.2% (Rosner et al., 2021). Extrapolated to a Swedish context, this would correspond to approximately 126,000 people. To our knowledge, evidence-based treatments for PGD are not widely available in Swedish routine care, and comprehensive international treatment guidelines are still emerging.

A growing body of research supports the efficacy of internet-delivered and face-to-face cognitive-behavioural therapy (CBT) in alleviating symptoms of PGD, PTSD and depression after loss (Komischke-Konnerup et al., 2024; Srivastava et al., 2025; Zuelke et al., 2021). However, several limitations remain, including small sample sizes and a lack of long-term follow-ups. Furthermore, there are considerable differences between studies in which CBT components have been offered to participants (e.g., cognitive restructuring and/or exposure, and/or behavioural activation, as well as combinations with non-CBT interventions). This variation in treatment content across studies reduces the generalisability of findings. Significant differences have been observed between CBT and active control conditions in PGD symptom reductions (Boelen et al., 2007; Bryant et al., 2024; Shear et al., 2005; Rosner et al., 2025), suggesting that CBT mechanisms drive therapeutic change in treatments for bereaved. However, studies on grief-focused internet-delivered CBT (ICBT), and especially studies comparing grief-focused ICBT to active control conditions, remain scarce.

The current intervention is based on a grief-focused face-to-face CBT treatment, aiming to reduce avoidance behaviours, create a narrative about the loss, and challenge negative beliefs related to identity, life after loss and grief (Boelen et al., 2007), using cognitive restructuring and exposure exercises. The face-to-face treatment reduced symptoms of PGD and resulted in greater clinical improvements than twelve sessions of supportive counselling (Boelen et al., 2007). An internet-delivered version, which also includes behavioural activation (Eisma et al., 2015; Papa et al., 2013), has been developed from the face-to-face treatment and was evaluated in two trials: one therapist-guided (Lenferink et al., 2023) and one unguided version (Reitsma et al., 2023). Hence, this grief-focused ICBT is one of few treatments that have been evaluated in more than one trial and demonstrated reductions in symptoms of PGD, PTSD and depression. However, these studies applied broad inclusion criteria, encompassing clinically relevant symptom levels of PGD, and/or PTSD, and/or depression (Lenferink et al., 2023; Reitsma et al., 2023). In addition, one study (Reitsma et al., 2023) evaluated the treatment as an early intervention before the six-month time criterion for a formal ICD-11-based (and the 12 months for the DSM-5-TR-based) PGD diagnosis had been met. Furthermore, some previous trials have focused on specific subgroups of bereaved, including bereaved by traffic accidents (Lenferink et al., 2023) and during the COVID-19 pandemic (Reitsma et al., 2023). There remains a need to replicate and extend these findings in participants with PGD and diverse loss characteristics.

The recognition of PGD as a formal clinical condition highlights the need for effective and evidence-based treatments, that can support individuals struggling with persistent grief, regardless of the cause of death or population studied. Although the evidence-base supporting the efficacy of ICBT for PGD is increasing, some previous studies have assessed treatments for specific subgroups, such as bereaved siblings (Wagner et al., 2022a), bereaved by suicide (Treml et al., 2021; Wagner et al., 2022b), cancer (Kaiser et al., 2022), and traffic accidents (Lenferink et al., 2023). There is a need for more studies with larger and broader sample sizes in heterogenous samples of bereaved. This study is among the first to test the feasibility, acceptability, and potential efficacy of grief-focused ICBT for bereaved individuals, regardless of kinship to the deceased and cause of death, conducted in a clinical setting resembling routine care. It is also one of the first to examine participants' experiences of undergoing grief-focused ICBT through post-treatment interviews.

The primary aim of the study was to evaluate the feasibility and acceptability of grief-focused ICBT for PGD in bereaved adults in a Swedish context. A secondary aim was to assess the potential effect of the treatment on symptoms of PGD, PTSD and depression.

## 2 Material and methods

### 2.1 Design and setting

The study used a longitudinal, uncontrolled, multiple-methods design, combining self-reported measurements at pre-, post-treatment and 4-month follow-up, as well as post-treatment interviews. The study was conducted at a publicly funded outpatient healthcare centre in Uppsala, Sweden. The healthcare centre offers internet-delivered, text-based CBT interventions, including depression, generalised anxiety disorder and sleep difficulties.

### 2.2 Procedure

**Fig. 1:**
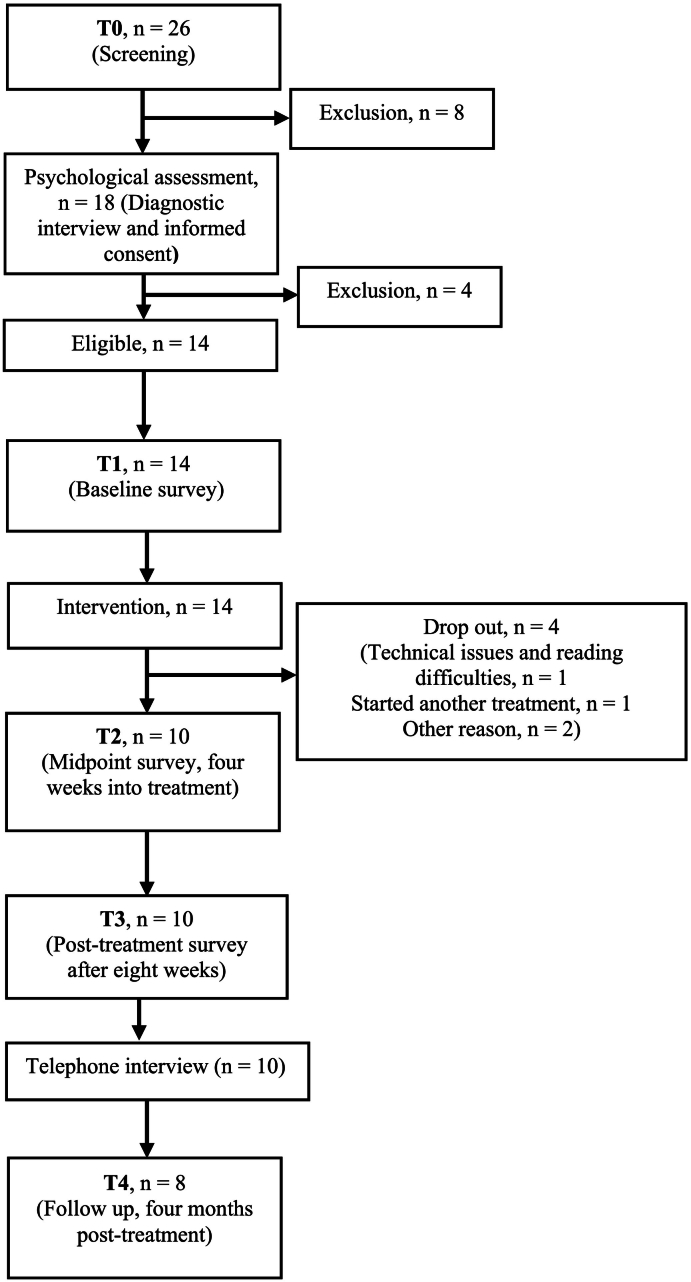
Flowchart of the study.

Fig. 1 illustrates timepoints for data collection and participant flow through the study. Enrolment began in September 2022 and ended in December 2022. Participants were recruited when seeking healthcare in Uppsala region as well as through advertisements on social media and registered through self-referral. Interested applicants completed an online screening questionnaire. The screening included self-reported symptoms of prolonged grief according to the *Traumatic Grief Inventory Self-Report Plus* (TGI-SR+) (Lenferink et al., 2022; Lenferink et al., 2024), targeted questions to screen for inclusion and exclusion criteria, and questions about demographic information.

Participants were excluded if they had other ongoing psychological treatment, other comorbidities that could have prevented the implementation of treatment or that required more urgent care (e.g., severe anxiety or depressive disorders, bipolar disorder, personality disorders, psychosis or high suicidality), if they had changed their dose of medication for mental symptoms in the past four weeks, or planned to do so in the coming two months. Individuals subjected to ongoing domestic violence were assessed and, if necessary, excluded from the study and referred to appropriate support services in accordance with the standard procedures of the healthcare centre. After initial screening of eligibility criteria, participants went through a psychological assessment by telephone or videoconferencing with a licensed psychologist at the healthcare centre. During the psychological assessment, the diagnostic interview *Mini International Neuropsychiatric Interview* (M.I.N·I) (Sheehan et al., 1998) was administered to screen for the most common psychiatric disorders. The psychologist asked about each PGD criteria as per ICD-11, to determine whether the diagnostic criteria were met. Participants who did not meet the diagnostic criteria for PGD were excluded.

Eligible participants were given access to the treatment after the psychological assessment and the baseline questionnaire were completed. Regular patient fees were charged for the psychological assessment according to the standard routines of the recruitment site. Applicants who did not meet eligibility criteria were contacted via telephone and referred to their primary healthcare provider. The intervention was cost-free for all participants.

### 2.3 Participants

Adults who lost a family member, a partner/spouse or a close friend at least six months ago, reported symptoms of PGD as per ICD-11, were residents of Uppsala region and could understand, write, and speak Swedish were eligible to participate in the study. A cut-off of ≥71 on the TGI-SR+ (Lenferink et al., 2022; Lenferink et al., 2024), measuring PGD symptoms, was used as a reference point for probable PGD. However, participants whose scores fell below the cut-off at screening were included if they were clinically judged to likely fulfil the criteria for PGD (i.e., endorsing the PGD core symptom criteria per ICD-11). No predefined threshold for how far below cut-off participants could score and still be considered for eligibility was specified.

### 2.4 Intervention

The treatment was accessed via the platform “1177 Stöd och behandling” (“1177 Support and treatment”), a secure digital platform routinely used for delivering internet-based psychological treatments within the Swedish national healthcare system. The treatment is based on a face-to-face protocol of CBT for PGD, combining cognitive restructuring and exposure exercises (Boelen et al., 2007) with components of behavioural activation (Eisma et al., 2015; Papa et al., 2013). The internet-delivered version of the treatment, developed for individuals bereaved by traffic accidents (Lenferink et al., 2023; Lenferink et al., 2020), was translated into Swedish. The treatment content was adapted to support bereaved regardless of the cause of death by using examples and case illustrations describing different kinds of deaths and losses. Table 1 shows an overview of the treatment. The treatment comprised eight modules delivered over an eight week-period. Modules were released weekly on a fixed day, provided that the psychologist judged the participant had completed the core components of the module.

**Table 1:** Content of the Internet-based Cognitive Behavioural Treatment (ICBT).

Module	Description
1: Introduction	Psychoeducation about grief, common grief reactions and factors that may facilitate or hinder the adaptation process. Rationale for and goals with the CBT treatment are introduced.
2: Exposure	Rationale for exposure exercises and reducing avoidance behaviours. Digital worksheets are used to plan and conduct the first in vivo exposure exercise (e.g., visiting the gravesite, hospital or the deceased's workplace).
3: Exposure	Written exposure exercises, where the participant describes the events leading up to the death and in-depth exposure of the moment of death.
4: Exposure	Looking at pictures and writing a letter to the deceased, where the participant in detail describes the consequences of the loss and what they miss with the deceased.
5: Cognitive therapy	Rationale for cognitive therapy. Digital worksheets are used to identify own negative thoughts and how these impact emotions and behaviour.
6: Cognitive therapy	Common thinking errors are introduced. Digital worksheets are used to identify own thinking errors. The participant challenges negative thoughts related to identity and other people in behavioural experiments.
7: Behavioural activation	Suggestions for potential meaningful daily activities are offered. Digital worksheets are used to plan and carry out meaningful daily activities and achieving future goals.
8: Summary and maintenance plan	The participant continues working with meaningful activities and planning future goals. The participant reviews their goals with the treatment and writes a maintenance plan. A final exercise is given where the participant writes a letter to the deceased about the future.

During the intervention, the participants read texts, answered questions and completed digital worksheets for homework assignments for each module. The digital worksheets were used to plan and execute homework assignments, such as behavioural experiments, behavioural activation, written exposure, and cognitive restructuring exercises. Feedback was given through asynchronous online text messages sent on the treatment platform. The therapists were instructed to spend approximately 30 min per week providing feedback based on the participants' digital worksheets. The purpose of the therapist feedback was to support participants, reinforce their engagement, validate emotional responses and help them to overcome possible difficulties encountered during treatment. This included providing clarifying instructions on homework assignments, answering questions about the treatment content, assisting with problem-solving homework assignments and acknowledging participants' efforts.

The participants and therapists could communicate through text messages on the treatment platform or, if needed, by telephone. The therapist contacted participants over the phone if they showed signs of deterioration, suicidal ideation or requested additional support. The number of telephone calls was not recorded in this study.

### 2.5 The therapists

The therapists were two licensed psychologists at the healthcare centre with one- and four-years' experience of working with ICBT but were not experts in PGD. One of the therapists (EH) is a co-author of this study. Before study onset, the therapists received training by MB and JS. Supervision and guidance were offered during five prescheduled sessions and additional supervision was offered upon request.

### 2.6 Ethical considerations

Written informed consent was obtained in the treatment platform before the baseline assessment. The study has received ethical approval from the Swedish Ethical Review Authority (registration ID 2022-02707-01).

Suicidal risk was monitored weekly during treatment using the *Montgomery-Åsberg Depression Rating Scale Self-Report (*Svanborg and Åsberg, 2001*)* question nine, which assesses suicidality. Participants who scored four or more on this item were contacted by telephone by a psychologist for further assessment of suicide risk. If necessary, participants were referred to the emergency psychiatric unit and/or outlined an individualised safety plan in collaboration with their psychologist.

Participants who showed signs of deterioration during treatment were contacted by their psychologist by telephone for further support.

## 3 Outcome measures

All data in the study were collected through the treatment platform. Participants who dropped out were not administered post-treatment or follow-up questionnaires, as they discontinued their participation in the study.

### 3.1 Feasibility

The feasibility of the treatment was assessed by the screening-to-enrolment ratio, reasons for exclusion, as well as therapist time spent on delivering the treatment, measured by the time spent on providing feedback per module, and number of messages sent from therapists to patients.

### 3.2 Acceptability

The acceptability of the treatment was assessed through dropout rates from the study, treatment engagement, satisfaction with the treatment, beliefs in treatment efficacy and credibility, and occurrences of adverse events.

Dropout was defined as active discontinuation from the treatment, by informing the psychologist that they did not wish to continue with the treatment. Participants who became inactive (e.g., did not log into the treatment platform) for more than one week were contacted by their psychologist either online or via telephone. Participants who could not be reached and remained inactive after several contact attempts were also considered dropouts.

Participants' engagement was measured by the number of completed modules and by self-reported assessments of their own work with the treatment. A module was considered complete if the participant had read the material and completed its core components.

Treatment satisfaction was measured by twelve questions administered at post-treatment (Table 2) inspired by the *Client Satisfaction Questionnaire 8* (CSQ-8) (Attkisson and Zwick, 1982). Examples of survey items included “Overall, I am satisfied with the treatment I received” and “It worked well to receive treatment via the internet”, answered on a 1–4 Likert scale (“do not agree at all” - “agree completely”).

**Table 2:** Participants' views of the treatment: survey questions (*n* = 10).

	Do not agree at all	Agree to some extent	Mostly agree	Completely agree
Overall view of the treatment				
Overall, I am satisfied with the treatment I received	1	–	3	6
The treatment has helped me adopt a better approach to my problems	–	1	7	2
My most inhibiting symptoms have decreased due to the treatment	–	2	6	2
I would recommend the treatment to someone else in a similar situation	–	–	3	7
The treatment met my needs	–	3	5	2

Treatment material				
I am satisfied with the quality of the treatment's modules	–	–	7	3
Overall, I received good help from the material in the treatment (i.e., everything except my personal contact with my therapist)	–	–	7	3
The text felt interesting and relevant to read	–	–	5	5
It was easy to understand how to work with the treatment and homework assignments	–	3	5	2
It worked well to receive treatment via the internet	–	–	7	3

Therapist contact				
The contact with my therapist helped me in my work with the treatment	–	–	2	8
It feels important to have the same therapist throughout the treatment	–	–	1	9

To measure participants' beliefs in the efficacy and credibility of the treatment, an adaptation of the *Treatment Credibility Scale* (TCS) (Borkovec and Nau, 1972; Devilly and Borkovec, 2000) was administered the second and fifth week of treatment. Participants were asked to rate their belief in the credibility and perceived efficiency of the treatment on a 5-item scale. Answers ranged from 1 to 10 and total scores from 5 to 50, where higher scores indicate higher beliefs in treatment efficacy and credibility. The TCS does not have a specific cut-off for determining treatment credibility, however, the scale can be used to compare different treatments. Examples of survey items include “How logical does this type of treatment seem to you?” and “How confident are you that this treatment will be successful in reducing your symptoms?”

Adverse events have previously been defined as negative changes in patients' symptoms, functioning or other unwanted events experienced during treatment, while serious adverse events refer to severe negative outcomes such as hospitalisation, suicidality or enduring deterioration or impairment (Klatte et al., 2025). In the current study, adverse events were assessed retrospectively through a self-reported measure at post-treatment. Participants could answer multiple times in case they had experienced several adverse events (e.g., “During the course of treatment, have you experienced any undesirable events that you believe are related to the treatment or any unwanted effects from the treatment?”, “Describe the undesirable event or unwanted effect. Also mention when these events/effects occurred during the treatment, how often they occurred and for how long each event/effect lasted.”)

### 3.3 Experiences of treatment

Participants' experiences and opinions about the treatment were investigated through semi-structured telephone interviews (see Supplementary material for the interview guide). JS and YR (YR, a registered nurse and psychotherapist in training at the time of the study, supervised by JS) developed the interview guide for the present study, which was not pilot tested prior to use. Participants were asked whether they would be interested in participating in a post-assessment interview, and those who consented were contacted after treatment completion. One interview had to be repeated due to technical issues during the initial recording. The interviews were conducted by one of the authors (YR). The interviews were conducted on average 32 days post-treatment (median: 29 days, range: 7–86 days post-treatment). The interviews lasted between 15 and 50 min, were audio-recorded, and transcribed verbatim. All participants who consented were interviewed.

### 3.4 Changes in self-rated symptoms

Surveys measuring symptom severity were administered at baseline, post-treatment, and follow-up at 4 months post-treatment.

PGD symptoms were measured using the self-assessment scale TGI-SR+ (Lenferink et al., 2022; Lenferink et al., 2024), which consists of 22 questions with answers ranging from “Never” to “Always” and total scores from 22 to 110. Participants were instructed to rate the presence of PGD symptoms during the preceding month. A cut-off of ≥71 can be used to discern probable PGD in the Swedish version of the TGI-SR+ (Lenferink et al., 2024).

Symptoms of loss-related PTSD were measured using the self-assessment scale Posttraumatic Stress Disorder Checklist for DSM-5 (PCL-5) (Blevins et al., 2015), which consists of 20 questions with answers ranging from “Not at all” (0) to “Extremely”. Participants were asked to rate the presence of loss-related PTSD symptoms the preceding month. The instruction was changed from “the stressful event” to “your loss”. A cut-off of ≥30 was used to identify probable PTSD (Bondjers, 2020).

Depression was measured by the self-assessment scale Patient Health Questionnaire 9 (PHQ-9) (Kroenke et al., 2001), which consists of nine questions. Participants were asked rate the presence of depressive symptoms the two preceding weeks. Answer options range from “Not at all” (0) to “Almost everyday”. A cut-off score of ≥10 indicates probable depression.

## 4 Data analysis

Quantitative data on participants' views and experiences were summarised descriptively. IBM SPSS 28.0.1.0 was used for all statistical analyses. Wilcoxon paired samples test (2-tailed) was used to test differences in symptom change between baseline and post-treatment, and between baseline and follow-up. Cohen's *ds* were used to calculate within-group effect sizes between baseline and post-treatment, and between baseline and follow-up.

Conventional content analysis, in which codes, subcategories and categories are derived directly from the data, was used to analyse the interviews (Hsieh and Shannon, 2005). The transcripts were read several times independently by authors FB, RE and JS to develop an understanding of the context and gain a sense of the whole. Next, authors FB and RE coded the material independently. Thereafter, FB and RE discussed and compared their codes which were subsequently sorted into subcategories based on similarities and differences. A total of 32 subcategories were sorted into five categories.

## 5 Results

### 5.1 Demographic and clinical characteristics

Twenty-six participants registered their interest in the study, and 14 participants were included. Demographic characteristics of the participants are shown in Table 3. Thirteen participants were women. The most common loss was that of a sibling (*n* = 5) and mean time since loss was 3.65 years (SD = 4.26).

**Table 3:** Baseline characteristics and demographics.

	Total sample (n = 14)
Age (years)	
Mean (SD)	39.43 (15.17)

Gender	
Female	13
Male	1

Highest education	
University	10
Secondary school	4

Occupation	
Working	6
Student	3
Sickleave/partial sickleave	2
Unemployed	1
Pensioner	2

Martial status	
Married	2
Partner	6
Single	6

Deceased is…	
Parent/stepparent	3
Grandparent	1
Sibling	5
Spouse	4
Child	1

Months since death	
Mean (SD)	43.76 (51.13)
Range minimum, maximum	5, 142

Baseline symptoms (m, SD)	
TGI-SR+	77.21 (14.04)
PCL-5	38.79 (14.96)
PHQ-9	12.93 (6.20)

TGI-SR+ = Traumatic Grief Inventory - Self Report Plus; PCL-5 = Posttraumatic Stress Disorder Checklist for DSM-5; PHQ-9 = Patient Health Questionnaire 9.

Based on the diagnostic interview M.I.N·I, seven participants were diagnosed with major depressive disorder and three with PTSD. Of the ten participants who continued with the treatment, one had previous experience of ICBT, while nine had no previous experience.

No significant differences were found in PGD (*p* = .94), PTSD (*p* = .37) or depressive symptoms (*p* = .89) between dropouts and study completers at baseline (Table 4).

**Table 4:** Baseline symptoms of PGD, PTSD and depression in dropouts and study completers.

	Dropouts (n = 4)	Study completers (n = 10)
TGI-SR+		
M (SD)	77.00 (16.87)	77.30 (13.78)
Min – max	62–94	57–104
PCL-5		
M (SD)	40.50 (20.07)	38.10 (13.68)
Min – max	11–56	19–70
PHQ-9		
M (SD)	11.75 (8.46)	13.40 (5.54)
Min – max	3–19	6–24

TGI-SR+ = Traumatic Grief Inventory - Self Report Plus; PCL-5 = Posttraumatic Stress Disorder Checklist for DSM-5; PHQ-9 = Patient Health Questionnaire 9.

### 5.2 Feasibility

#### 5.2.1 Eligibility

Twenty-six applicants completed screening, of whom twelve participants were excluded based on eligibility criteria. The most common reason for exclusion was that some other mental health issue than PGD was considered to be the main reason to seek treatment (Table 5).

**Table 5:** Reasons for exclusion before and after the psychological assessment (*n* = 12).

Reasons for exclusion	n
Something other than prolonged grief was assessed to be the main issue (e.g., PTSD).	3
Other contact was recommended, due to ongoing crisis not associated with the loss	2
High suicidality or ongoing contact with specialist psychiatry	2
No need for treatment (i.e., subclinical symptoms)	1
Insufficient information available from the registration/did not schedule an appointment for psychological assessment	1
Begun other treatment	1
Other/unknown	2

Information about how participants were recruited was available for eleven participants in the study (ten completers and one dropout). Four were referred by a clinician or psychologist, six found the study online, and one was recommended by an acquaintance. The participant who dropped out was referred by a psychologist while on a waitlist for another treatment and dropped out once that treatment became available.

#### 5.2.2 Therapist time and number of messages

The average time therapists spent on giving feedback per week and participant was 26.23 min (SD = 13.42). The most time was spent on Module 7 (behavioural activation and increasing meaningful activities, M = 53.38 min, SD = 13.17) and the least on Module 2 (in vivo exposure exercises, M = 19.67 min, SD = 7.40). On average, the therapists spent 148.00 min (SD = 86.13) in total per patient (range: 10–253, *n* = 14), including participants who dropped out or did not complete all eight modules. Among those who remained in treatment and where data was available for all modules, the total therapist time spent on providing feedback was on average 217.43 min (SD = 25.90, range: 168–253, *n* = 7). The average number of messages sent from therapists to participants (*n* = 13) was 17.54 (SD = 7.98). Most messages from therapists to participants consisted of weekly encouragement and longer feedback on homework assignments.

### 5.3 Acceptability

#### 5.3.1 Treatment engagement and dropouts

The average number of completed modules was 5.57 (SD = 3.30, n = 14) of eight. Of the 14 participants who were given access to the treatment, four dropped out before completing module 2 (29%). Ten participants (71%) completed at least four modules (i.e., half of the treatment), and eight participants (57%) completed all modules. This indicates that most participants, aside from those who dropped out (*n* = 4), worked with exposure exercises. The average number of messages sent from participants to therapists was 7.15 (SD = 5.18).

Ten participants completed post-assessment, and eight participants completed the follow-up after four months. In the post-treatment survey, all participants with available data (*n* = 10) responded that they had worked actively with most (*n* = 7) or all (*n* = 3) homework assignments and accessed the treatment at least twice a week. Seven participants estimated that they spent between 30 min and three hours per week working actively with the treatment. Three participants reported that they spent between four and six hours a week working with the treatment.

#### 5.3.2 Treatment experience and satisfaction based on the surveys

In Table 2, participants' satisfaction with the treatment, its material and the contact with the therapist is summarised. Most participants were satisfied with the treatment they received, while one participant was not satisfied at all. All would recommend the treatment to someone else in a similar situation. All participants found the contact with their therapist to be an important part of the treatment.

#### 5.3.3 Treatment credibility

The TCS measures participants' beliefs in treatment efficacy and credibility. The mean total score of TCS was 38.50 (SD = 6.31, range = 28–47, *n* = 10) the second week of treatment, and 37.60 (SD = 7.12, range = 30–50, n = 10) the fifth week.

#### 5.3.4 Adverse events

Five participants (50%) responded that they had experienced at least one undesired event or effect (Table 6). One participant explained that she had experienced more flashbacks and symptoms because of the treatment. Two participants described decreased well-being, of whom one had stayed at home for several weeks and reported being unable to eat or sleep well. One participant had felt increased yearning for the deceased and had cried whilst completing homework assignments. Another participant described crying for hours. The participant who reported increased symptoms and flashbacks still felt affected by this at post-treatment.

**Table 6:** Participants' experiences of undesired events (n = 10).

	Yes	No
Have you experienced any undesired event or unwanted effects due to the treatment?	5	5
Have you experienced any additional undesired events or effects?	0	10


	Not at all	Little	Moderately	Very much	NA
How negatively did the event affect you at the time?	1	1	2	0	1
How much does it affect you today?	3	0	1	0	1

### 5.4 Experiences of treatment

Ten post-treatment interviews were included in the results. All participants were female. In the qualitative analysis of the interviews, five categories presented below were found to represent the participants' experiences of the treatment. Table 7 shows the categories with examples of subcategories, codes and meaning units.

**Table 7:** Categories derived from the conventional content analysis with examples of subcategories, codes and meaning units.

Category	Subcategory	Code	Meaning unit
The treatment led to improved well-being, increased knowledge of own grief reactions and helped participants develop new approaches to grief and life after loss	The treatment has provided increased insight and understanding of oneself and one's own grief and emotional reactions	Started to feel that it's not just me Many who grieve feel guilt	“That you mentioned this about guilt, then I started to feel that, okay, but it isn't just me who experience guilt but many who feel grief or grieve feel guilt.”
Therapist-guided ICBT for prolonged grief had several advantages, and at the same time evoked a lot of emotions and could be difficult at times. Participants have several suggestions for improvement	There were times during treatment when participants felt worse and the treatment was emotionally heavy	Pretty difficult in the meantime Brings up a lot of memories you'd rather not think about	“Of course, it's been pretty difficult in the meantime, that it brings up a lot of memories that you'd rather not think about.”
Module 1–4 were emotional, but helped participants reduce avoidance and approach their grief	Module 4, describing who died and writing a letter to the deceased, evoked strong emotions among the participants but also provided new insights, an overview, and a sense of control over their grief	More structured Could put a label on what it is that is empty in everyday life	“It became more structured in a way then, that I really could put a label on what it is that is so incredibly empty in everyday life.”
Module 5–6 provided new perspectives on and insights about thoughts, while also being very difficult to understand and apply	Modules 5–6 were, according to participants, the most difficult part of the treatment. It was difficult to approach new perspectives about thoughts, describe thoughts and differentiate between thoughts and feelings	Everything has been empty Have not thought anything Difficult to think deeply and in a different way	“I've been more that everything's been empty. I haven't thought anything during these years. To change from the emotional exposure, to think rather deeply and in a different way, I thought this was… this was pretty difficult actually.”
Modules 7–8 facilitated many in getting started with activities and planning for the future	It was helpful to plan for the future, give advice to others in a similar situation and consider what to do or have in mind if one feels worse in their grief again. There was a sense of security, having a summary moving forward	Did not know I was capable of giving such good advice	“I didn't even know that I had… that I was capable of giving such good advice.”

#### 5.4.1 The treatment led to improved well-being, increased knowledge of own grief reactions and helped participants develop new approaches to grief and life after loss

The treatment increased participants' understanding of themselves, their grief, and how to manage grief reactions. One participant described it as, “*I have a new belief in myself, that I've got this. That ‘even if is really dark and it feels like rock bottom, I'll get through this.’”* (Participant 1).

The treatment challenged participants' negative beliefs about their identity and their future after the loss. Participants found new strategies for managing negative thoughts and intrusive memories: *“I can kind of almost ask [the intrusive images] to disappear. So I can manage them in a completely different way than before.”* (Participant 2) Participants also reported that the treatment reduced avoidance of loss- and grief-triggering reminders. One participant reflected that *“this was the first time that I could say out loud that, okay, she is not coming back.”* (Participant 3).

The treatment helped participants reengage in meaningful activities and create a more meaningful leisure time. One participant put it as *“I've gotten my life back on track because of this.”* (Participant 2).

#### 5.4.2 The online treatment had several advantages and evoked a lot of emotions

The internet format and the flexibility were appreciated by several participants: *“It was really good that one could do [the treatment] at home, in one's safe environment.”* (Participant 1) A suggestion to make the treatment more accessible was given by one participant: *“It would have been so nice to just have a button and press play and listen and think at the same time.”* (Participant 4).

Receiving online support and feedback from a licensed psychologist were important for several participants and facilitated their work with the treatment. One participant found this to be one of the most important aspects of the treatment: *“It felt like they were actually invested and could see things that I hadn't thought about.”* (Participant 5) While many valued the online format, some participants wished for at least one in-person meeting with their therapist.

Case examples of other bereaved people's experiences were helpful in identifying own emotions and thoughts. However, some participants missed examples that felt applicable to them: *“I didn't really find an example that I could relate to. […] And then I didn't really know what exercises I could do.”* (Participant 5) Participants also felt confusion regarding why certain parts, such as behavioural activation, were included in the treatment and how this would improve their grief. Some participants felt ambivalence about changing thoughts and behaviours*: “I thought at first that I want to keep [the negative thoughts], because it is negative, and the world is unfair. That's how it is and I don't want to look at it in a positive way.”* (Participant 3).

Working with one module per week was feasible according to some participants. Others found the second half of the treatment increasingly stressful as the assignments became more complex. One participant observed: *“Maybe, for [cognitive therapy and behavioural activation], it would be better with one and a half week, so that you actually manage to complete them*.” (Participant 5).

There were times during treatment when participants felt worse and the treatment was emotionally heavy. Some participants reported experiencing little difference in well-being between before and after treatment: *“I'm still in the midst of grief, I can't say this has really helped me one hundred percent.”* (Participant 4).

#### 5.4.3 Exposure exercises were emotional and helped participants reduce avoidance and approach their grief

The exposure exercises helped participants express their grief, organise their thoughts about the loss, and provided new perspectives on grief and loss. *“This was one of the most important parts for me, to be able to get a hold of everything that happened. This helped me sort things out, by talking about it and looking back at everything.”* (Participant 2).

Exposure to the moment of death was a challenging exercise which many participants previously had avoided thinking about. After completing the exercise many felt relief. One participant explained: *“I hadn't thought [about the event] in detail or, so much in depth, exactly what happened and what I felt.”* (Participant 3) Visiting a location associated with the deceased was also a challenging but appreciated part of the treatment, which disproved participants' negative assumptions. One participant visited her mother's grave for the first time: *“It turned out that what I had imagined before was completely wrong.”* (Participant 2).

The exercises where participants described their loss and wrote a letter to the deceased provided an overview and a sense of control over the grief. One participant reflected: “*It became more structured in a way then, that I really could put a label on what feels so incredibly empty in everyday life.”* (Participant 5).

#### 5.4.4 Cognitive restructuring provided new perspectives about thoughts but was also difficult to understand and apply

Cognitive restructuring provided participants with insights about the impact of thoughts, and demonstrated how emotions can be influenced through identifying, challenging and reframing negative thoughts. It also helped participants become aware of their own unhelpful thoughts: *“Identifying these heavy thoughts that I've had for a long time, it felt really, really relieving.”* (Participant 6) One participant shared how the treatment helped question negative assumptions in everyday life, *“You get to turn it around a bit. ‘How can I look at this in a different way?’”* (Participant 5).

This part of the treatment was the most difficult for many participants. It was difficult to apply new perspectives about thoughts and differentiate between thoughts and feelings. The cognitive distortions introduced in the therapy could also be confusing: *“This is where the modules became difficult, I think. I had a really hard time keeping track of what was what.”* (Participant 2).

#### 5.4.5 Behavioural activation and the maintenance plan facilitated many in getting started with activities and planning for the future

Behavioural activation has encouraged many participants in starting activities. One participant shared how making goals and planning activities made them seem less daunting: *“I realised […] there is nothing that's really stopping me. It's just to do it. So, it became a more nuanced way of thinking about these meaningful activities.”* (Participant 5) Other participants found it difficult to plan and carry out goals practically, with some stating that they already had attempted to engage in activities on their own: *“This was pretty unnecessary for my sake really. Because I have always done stuff.”* (Participant 7).

Several participants valued writing the letter where they summarised their insights from the treatment and outlined plans if they were to feel worse in the grief again. One participant described: *“I was so proud of my text, I thought ‘wow, what a survival letter!’ […] That's where I understood the impact of this study, how much this has helped me.”* (Participant 4).

### 5.5 Changes in self-rated symptoms

Total mean scores and SDs of self-rated symptoms of PGD, PTSD and depression, as well as effect sizes, are shown in Table 8. Wilcoxon paired samples tests indicated that symptoms of PGD (z = −2.70, *p* = .007), PTSD (z = −2.81, *p* < .001) and depression (z = −2.25, *p* = .02) decreased from baseline to post-treatment in the completers' sample (*n* = 10). Reductions were also observed between baseline and follow-up (*n* = 8) in symptoms of PGD (z = −2.11, *p* = .04), PTSD (z = −2.38, *p* = .02) and depression (z = −2.32, *p* = .02).Strong effect sizes were observed in symptoms of PGD (*d* = 1.01; 95% CI: 0.30—2.33) and depression (*d* = 1.11; 95% CI: 0.22—2.44) from baseline to post-treatment, as well as from baseline to follow-up for PGD (*d* = 1.18; 95% CI: 0.32—2.69), and depression (*d* = 1.07; 95% CI: 0.42—2.55). For PTSD symptoms, a medium effect size was found from baseline to post-treatment (*d* = 0.75; 95% CI: 0.53—2.04), and a large effect size from baseline to follow-up (*d* = 1.14; 95% CI: 0.35–2.64).

**Table 8:** Observed self-rated symptoms and effect sizes for the completers' sample.

	Baseline (n = 10)	Post-treatment (n = 10)	Cohen's d (CI-95%) pre vs post	Baseline (n = 8)	Follow-up (n = 8)	Cohen's d (CI-95%) pre vs follow-up
	M (SD)	M (SD)		M (SD)	M (SD)	
TGI-SR+	77.30 (13.78)	60.30 (19.28)	1.01 (0.30–2.33)	76.38 (15.47)	55 (20.31)	1.18 (0.32–2.69)
PCL-5	38.10 (13.68)	25.70 (18.83)	0.75 (0.53–2.04)	39.13 (14.86)	20.50 (17.60)	1.14 (0.35–2.64)
PHQ-9	13.40 (5.54)	7.30 (5.46)	1.11 (0.22–2.44)	13.50 (6.07)	7.25 (5.65)	1.07 (0.42–2.55)

TGI-SR+ = Traumatic Grief Inventory - Self Report Plus; PCL-5 = Posttraumatic Stress Disorder Checklist for DSM-5; PHQ-9 = Patient Health Questionnaire 9.

## 6 Discussion

This study aimed to evaluate the feasibility and acceptability of therapist-guided ICBT for PGD in Sweden, a context where no such evaluation has previously been conducted. Furthermore, it is among the first studies to systematically evaluate ICBT for PGD in a sample of bereaved individuals with diverse loss characteristics, in a clinical setting resembling routine care, and to investigate participants' experiences through post-treatment interviews. Findings suggest that ICBT is a feasible and acceptable treatment alternative for Swedish bereaved adults, with the potential of increasing the access to treatment for the patient group. Moreover, the treatment demonstrated preliminary effects in reducing symptoms of PGD, PTSD and depression.

Therapist time spent on delivering the treatment was lower (M = 3.62 h, SD = 0.43, *n* = 7) than in the face-to-face version, which consists of twelve 45-min sessions (Boelen et al., 2007). This suggests that ICBT could be a time-efficient option for improving access to PGD treatment. However, some modules required more time than the instructed 30 min. The format and amount of therapist guidance offered in other ICBT treatments for PGD have varied, including weekly 90-min group video calls with a therapist (Wagner et al., 2022b), weekly telephone calls (Tur et al., 2022) and written feedback on homework assignments (Eisma et al., 2015; Treml et al., 2021; Kaiser et al., 2022). Other ICBT treatments for social anxiety and depression have reported lower total therapist time (M = 2.33–2.48 h, SD = 1.57–2.00) (Hedman et al., 2014; El Alaoui et al., 2015) than those observed in this study, indicating that the present treatment may be more time-consuming than some other ICBT treatments. Notably, grief-focused ICBT for PGD has also shown efficacy in a self-guided format without therapist support (Reitsma et al., 2023).

All dropouts in this study occurred in the early phases of the treatment (before completion of module 2), suggesting that dropouts may be related to poor initial fit between participants' expectations, treatment preferences and the treatment they received. Another possible explanation could be that exposure exercises were perceived as intimidating and discouraged participation. However, the reasons for dropout were not systematically collected in this study. The dropout rate (28.6%) in the present study is lower than in previous trials of the online treatment, reporting dropout rates of 42.0% (Lenferink et al., 2023) and 40.6% (Reitsma et al., 2023), respectively. Of the remaining participants, several did not complete all modules within the eight-week treatment period, and some reported that the modules felt increasingly difficult and time-consuming. The average number of completed modules was 5.57 (SD = 3.30, *n* = 14), which corresponds to 68% of modules completed. This is similar to previous trials on the treatment, where participants on average completed 5.67–5.88 of eight modules (SD = 2.50–3.31) (Lenferink et al., 2023; Reitsma et al., 2023). Other trials on grief-focused ICBT for PGD have observed similar completion rates (72–77%) (Wagner et al., 2022b; Tur et al., 2022), as well as ICBT trials for other psychological disorders conducted in routine care (61–72% completed modules) (Hedman et al., 2014; El Alaoui et al., 2015; Etzelmueller et al., 2020; Hedman et al., 2013).

Participants generally found the treatment to be credible and relevant to their needs. Beliefs in efficacy and treatment credibility according to the TCS were relatively high (M = 38.50, SD = 6.31 week two of treatment, and M = 37.60, SD = 7.12 week five, *n* = 10), which is comparable to other internet-delivered treatments for various psychological conditions (M = 28.5–39.6, SD = 5.6–8.4) (Axelsson et al., 2020; Carlbring et al., 2005; Kaldo et al., 2008; Rozental et al., 2024). This suggests that participants in the current study found the intervention credible and effective. Other ICBT trials for PGD have also reported high satisfaction and usability rates among participants (Eisma et al., 2015; Tur et al., 2022).

Few previous studies on ICBT for PGD have investigated whether interventions are associated with adverse events. One trial on an ICBT programme for PGD found that 50% of participants had experienced the intervention as aversive or unpleasant to at least some extent (Tur et al., 2022), although the exact reasons for this were not collected. In this study, 5 (50%) of participants reported adverse events, mainly related to reduced well-being and increased symptoms. One participant reported that the adverse event was still ongoing at post-treatment, indicating that although most adverse events appeared to be temporary, some may persist beyond the treatment period. Temporary increases in distress or symptoms may be expected during exposure to loss reminders (Boelen et al., 2007). However, the high proportion of participants reporting adverse events in this study underscores the importance of systematically monitoring and assessing the nature and seriousness of adverse events in future studies of grief-focused ICBT, to determine whether the intervention should be modified in terms of content and/or structure. In addition, these findings emphasise the importance of adequately preparing participants that they will be asked to confront emotional and potentially upsetting memories and offering support during these modules.

In the post-treatment interviews, exposure exercises emerged as a valued and central component of the treatment, which helped participants confront avoided memories and places. This aligns with the theoretical understanding of avoidance as a key maintaining factor in PGD (Boelen et al., 2007; Boelen et al., 2006) and previous research which highlights exposure as one of the core components in effective PGD treatments (Komischke-Konnerup et al., 2024). In contrast, participants' responses to behavioural activation were more varied. Some participants highlighted it as one of the most important parts of the treatment, and others found it irrelevant or difficult. One previous randomised controlled trial (RCT) comparing internet-based exposure to behavioural activation (Eisma et al., 2015) found higher dropout and lower satisfaction rates in the group receiving behavioural activation. These findings suggest that behavioural activation may be associated with challenges related to participant engagement and a lack of perceived relevance to participants' problems. Possible reasons may be that participants' motivation to undertake behavioural activation exercises is lower in online settings, or that the association between behavioural activation and PGD symptoms was not made sufficiently clear to participants.

The current study observed medium to large within-group effect sizes in symptom reductions between baseline and post-assessment, which were maintained at four-month follow-up. These effects are broadly consistent with previous studies on CBTs for PGD, in both online and face-to-face formats (Komischke-Konnerup et al., 2024; Srivastava et al., 2025; Zuelke et al., 2021). In previous trials of the online treatment, large within-group effect sizes were observed in PGD (*d =* 1.34–1.67), PTSD (*d =* 1.26–1.57) and depressive symptoms (*d =* 1.16–1.40) (Lenferink et al., 2023; Reitsma et al., 2023). Similarly, the present study observed effect sizes of *d =* 1.01 for PGD, *d* = 0.75 for PTSD and *d* = 1.11 for depression between pre- and post-treatment. However, as the present study was uncontrolled, had a very small sample, limited statistical power, and post-assessments were only administered to participants who continued with the treatment, these findings should only be considered preliminary and may overestimate treatment effects.

Future adaptations could focus on tailoring interventions to participants' specific needs, such as making modules optional. Some participants found the cognitive modules difficult to understand, suggesting that therapist guidance and clarifications might be especially important during cognitive exercises. The feedback from the participants in this study has been used to inform improvements of the treatment, including audio-recordings of the treatment material, clarifications and summaries in cognitive modules, and extending the length of the treatment from eight to ten weeks in preparation of an RCT.

The study has several limitations. Objective measures of participants' work with the treatment outside the digital platform were not collected, which means it is unclear to what extent participants actually engaged with the treatment in their everyday lives and completed its core components. For instance, the number of telephone calls between therapists and participants was not recorded, although this may be an important indicator of the level of additional support required and the feasibility of the treatment. Furthermore, feasibility and acceptability thresholds were not defined a priori, which should be considered when interpreting these outcomes. Participants who dropped out were not followed up, meaning that post- and follow-up assessments were only administered to participants who continued with the treatment. This might lead to an overestimation of effect sizes. In addition, the almost all-female sample limits generalisability to male patients, and the lack of a control group prevents us from attributing improvements solely to the treatment. Because the causes of the losses were not collected, we cannot draw conclusions about the relationship between outcomes and these characteristics of the sample, nor can we make specific claims about the extent to which the findings generalise to groups who experienced particular types of losses.

Notwithstanding these limitations, the current study is among the first to investigate participants' experiences of undergoing grief-focused ICBT for PGD using post-treatment interviews in a routine care setting, and to investigate the potential efficacy of ICBT irrespective of the cause of bereavement or relationship to the deceased.

Future research should compare ICBT for PDG to active control conditions, in order to assess whether observed improvements are due to common therapeutic factors, such as participants' expectations, placebo, and the support of a therapist, or whether they may be attributed to the CBT components. There is also a need for research looking into mediators and moderators of treatment effects (Komischke-Konnerup et al., 2024; Avis et al., 2025), to better understand the mechanisms behind therapeutic change in treatments for bereaved. In this way, the optimal conditions for treatment effectiveness may be identified.

In sum, the purpose of this study was to investigate the feasibility and acceptability of grief-focused ICBT for PGD in a Swedish context. The study suggests ICBT for PGD is feasible, acceptable, preliminarily effective and associated with low participant risk. Given the promising preliminary effects and the relatively low therapist time required, ICBT for PGD has the potential to improve access to care. A fully powered RCT is warranted to evaluate the efficacy of the current ICBT programme.

## Declaration of competing interest

The authors declare that they have no known competing financial interests or personal relationships that could have appeared to influence the work reported in this paper.
