# Supplementary material for: Feasibility, acceptability and potential effects of internet-based cognitive behavioural therapy for prolonged grief
Source: Internet Interv. 2026 Jun 17;45:100963. doi: 10.1016/j.invent.2026.100963 (PMC13284429; doi:10.1016/j.invent.2026.100963)
Supplement: Supplementary file 1 — Supplementary material 1 [file mmc1.docx]

# Supplementary material

## Interview guide

1. How come you participated in the study/chose internet-based therapy?
2. Do you have any prior experience of internet treatment?
3. Has the treatment led to any positive consequences? If yes, please elaborate
4. Has the treatment led to any negative consequences? If yes, please elaborate
5. The first part of the treatment, module 1-4, which is called Task 1 “To accept the loss and the pain that comes with it”, where you worked with exposure. Some perceive this part of the treatment as very emotional. How was it for you?
6. What did you think about these modules?
7. How was it to do the exercises, where you got to write about [what happened]?
8. In module 5-6, which is called task 2 “Trusting oneself, other people, life and the future”, the focus changed and you worked with thoughts and cognitions. How was it to change, from focusing on the loss and emotions to working more with thoughts?
9. How was it for you, to work with module 5-6, thoughts?
10. What did you think about these modules? How was it to do the exercises?
11. The last part, module 7-8, was to “To engage in helpful activities”, which is about doing more activities. How was it for you?
12. What did you think about these modules?
13. How was it to do the exercises?
14. Has anything been particularly good about the treatment?
15. Was any module particularly good?
16. Were any exercises particularly good?
17. Was something difficult in the treatment?
18. Was any module particularly difficult to do?
19. Were any exercises particularly difficult to do?
20. Do you miss anything in the treatment?
21. Can we do something to improve the treatment?
22. Was there enough time between the modules and tasks/assignments?
23. Have you done anything else since the study started, apart from the treatment, that you think could have affected your mood?
24. Is there anything you would like to talk about the treatment that I haven’t asked about?
25. (If the participant dropped out in advance): How come you ended the treatment in advance?
26. After the treatment, do you know how to take care of yourself to prevent relapse?
27. What can become future challenges for you, do you think?
28. Is there anything else that you’re thinking of?

Follow-up questions:

- Can you elaborate a bit more?
- Can you elaborate more when you say…?
- Can you give an example?
